# Editorial: Mitochondrial Biology and Its Role in Metabolic Diseases

**DOI:** 10.3389/fendo.2022.944728

**Published:** 2022-07-20

**Authors:** Jochen G. Schneider, Effie Tozzo, Manu V. Chakravarthy

**Affiliations:** ^1^ Luxembourg Centre for Systems Biomedicine (LCSB), University of Luxembourg, Belvaux, Luxembourg; ^2^ Saarland University, Medical Center, Department of Internal Medicine II, Homburg, Germany; ^3^ Avilar Therapeutics, Waltham, MA, United States; ^4^ Carmot Therapeutics, Berkeley, CA, United States

**Keywords:** interorgan crosstalk, insulin resistance, NAFLD, neurodegeneration, oxidative phosphorylation, energy homeostasis, metabolic flexibility

Starting with anecdotal reports there is now increasing evidence that chronic non-communicable diseases such as type 2 diabetes, obesity, cancer, and several neurodegenerative conditions may be interlinked by common pathobiological mechanisms. Mitochondria are prime candidates to modulate such intersecting processes because of their crucial role in energy homeostasis influencing nearly every tissue and organ of the body. Defects in mitochondrial metabolism can occur at all ages and produce a plethora of multiorgan symptoms, as illustrated through the research compiled for this Research Topic of *Frontiers in Endocrinology*.

While the primary role of mitochondria is in aerobic energy generation, the tricarboxylic acid cycle (TCA) they harbor serves as the central trafficking nexus not only for the supply of intermediary substrates to synthesize macronutrients, but also provides substrates for signaling processes controlling cellular function and fate (1, 2). The TCA cycle orchestrates the interplay of the major metabolic flux pathways – glycolysis, fatty acid synthesis/oxidation, pentose phosphate and serine pathways, inclusive of amino acid degradation, and integrates them with oxidative phosphorylation (**Figure 1**). This process is tightly titrated and metabolic flexibility is maintained by constant draining and replenishing of substrates (ana- and cataplerosis) in and out the TCA cycle.

**Figure 1:**
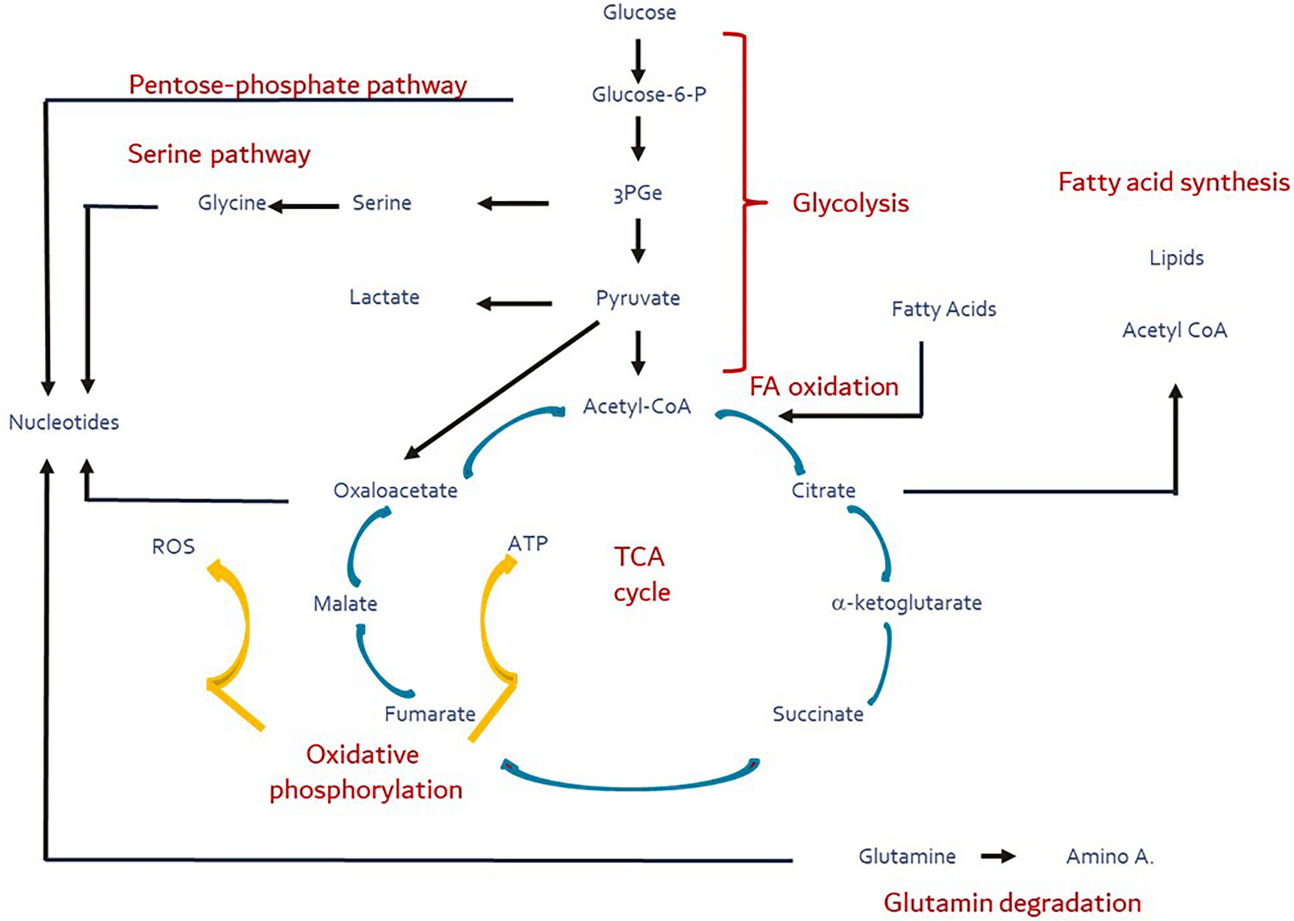
Major metabolic pathways (red) and their substrates and products (blue). Yellow arrows depict the oxidative phosphorylation during which ROS production is facilitated. These pathways are intertwined directly or indirectly *via* the TCA cycle (ana- and cataplerosis). Figure adapted and modified from Buck et al. (3).

Albhaisi and Sanyal characterize a “metabolic inflexibility” and its contribution to the development of the metabolic syndrome, non-alcoholic fatty liver disease (NAFLD) and non-alcoholic steatohepatitis (NASH). They highlight the dynamic interplay between liver, skeletal muscle and adipose tissue, and point to the importance of excess free fatty acid (FFA) availability driving insulin resistance. Liver insulin resistance produces a metabolic inflexibility to handle substrates. In turn, hepatic *de novo* lipogenesis is promoted, diverting citrate away from the TCA cycle (4). Together, these findings underscore inter-organ crosstalk as a central feature in insulin resistance (5), including NAFLD/NASH (Chakravarthy et al.). While lipotoxicity (6), mitochondrial dysfunction (7), endoplasmic reticulum (ER) stress and altered ER-mitochondria communication (8, 9) have all been ascribed as likely drivers of insulin resistance, it is also conceivable that insulin resistance as a general phenomenon could directly impact mitochondrial function and substrate handling in a cell-specific fashion. Dysregulation of the homeostatic balance of inter-organ axes by flooding it with excess substrates and/or an inability to handle substrate load could ultimately result in lipotoxic stress that involves mitochondrial dysfunction, inflammation, and ER stress (10). Further studies are warranted to deconvolute the mechanistic basis underlying the bidirectional axis of mitochondrial dysfunction and insulin resistance.

Metabolic inflexibility may also influence the physical interactions between mitochondria and the ER *via* the mitochondria-associated membranes (MAMs) that Cheng et al. focus in this review series. The signaling between mitochondria and the ER has shown to be dysregulated in type 2 diabetes (T2D), neurodegeneration, and obesity (11). MAMs may be key regulators that modulate the link between the two organelles by either phosphorylation events or specific localization of signaling proteins bridging the gap between mitochondria and the ER, or by facilitating Ca^2+^ exchange that regulates cytosolic Ca^2+^ content for signaling purposes and ATP production. Hence, ER-mitochondria miscommunication, possibly induced by insulin resistance, may result in metabolic diseases in a cell-autonomous fashion.

Mulica et al. take on this “cell-autonomous phenotype” by describing how the energy metabolism of neurons and astroglia is wired and how dysregulation of such homeostasis at the level of the mitochondria may result in neurodegeneration. Astrocytes support neuronal metabolic homeostasis and redox balance. This effect may result from a protective coupling of glycolysis and oxidative phosphorylation within the cells and likely also across cells. The astrocytes may sense and manage changes in neuronal glucose metabolism. This regulation may include coupling FFA metabolism between astrocytes and neurons and/or switching glutamate and glutamine towards differential use, such as neurotransmitter or as fuel to the TCA cycle, as appropriate. While neurodegeneration is accompanied by changes in glucose metabolism in neurons, how glucose precisely contributes to neurodegeneration remains challenging given the complexity of metabolic fluxes and compensatory phenomena. For instance, increases in ketone bodies have been found in patients with Parkinson´s and Alzheimer´s disease (12). They may exert antioxidant effects when glycolysis is decoupled from oxidative phosphorylation (13). Astrocytes may also charge the TCA cycle and ATP production through astrocyte-specific pyruvate carboxylase (14). The authors also describe differences in the mitochondrial complex chain in astrocytes and neurons that are coupled with transcriptional activity to balance out the metabolic and antioxidant state.

Pesta et al. delve into mitochondrial function and describe the *in vivo* and *in vitro* functional consequences of a single nucleotide polymorphism (SNP) in the NADH dehydrogenase-1ß subcomplex subunit 6 (*NDUFB6*) gene. NDUFB6 is a mitochondrial complex I protein that has been associated with exercise-related mitochondrial plasticity, insulin sensitivity, and ATP synthase flux (Pesta et al.). They show *in vivo* that the SNP in *NDUFB6* (*G* allele) changes physical exercise outcomes in subjects with diabetes. This effect, likely muscle-mediated, reduces mitochondrial respiration rate and insulin signaling. The study highlights the importance of maintaining the appropriate function of the mitochondrial complex chain and oxidative phosphorylation in metabolically demanding tissues (e.g., skeletal muscle), which in turn could impact whole-body metabolism to assuage metabolic diseases such as T2D and NAFLD/NASH. As altered functions of other complex I subunit genes (e.g., *NDUFA4, NDUFA9*) have been found in postmortem brain tissue from patients with Alzheimer´s disease (15), it seems conceivable that one may also identify *NDUFB6* SNPs in neurons/astrocytes and delineate its consequences (15). Thus far, no outcome studies of *NDUFB6* SNP carriers have been conducted.

Wang et al. describe the role of alpha-aminoadipic acid (2-AAA), a metabolite in the mitochondrial degradation pathway of the essential amino acid lysine to acetyl-CoA that ultimately can enter the TCA cycle. This provides a potential alternative pathway that may supply the TCA cycle with acetyl-CoA when glycolysis shunt/pyruvate dehydrogenase is blocked. The dehydrogenase E1 and transketolase domain containing 1 (*DHTKD1*) gene encodes the enzyme that produces 2-AAA (16); 2-AAA is shown to be associated with T2D (17). Mouse studies revealed a metabolic phenotype when *DHTKD1* is disrupted, yielding a phenotype that appears to be mediated by defective mitochondrial biogenesis and increased oxidative stress. Lack of mitochondrial function was compensated for by upregulation of mitochondrial biogenesis likely driven by signaling activity amplification. These findings along with the contributions from Mulica et al. and Wang et al. suggest that metabolic flexibility depends on structural integrity of the mitochondria space and its membranes.

More than 75 years after the seminal work of the Coris that represented one of the first descriptions of interorgan communication—conversion of lactate produced by anaerobic glycolysis in muscle to be recycled and converted to glucose by the liver, facilitating metabolic adaptation to energy demands (18)—we continue to unravel the subcellular and molecular machinery underlying energy homeostasis, with a spotlight on the mitochondria. This critical organelle has been ascribed to play major roles in cell differentiation, cancer, neurodegeneration, and metabolic diseases. Metabolic requirements of cells and organs are tightly regulated, and metabolic flexibility is supported by multiple redundant mechanisms. In-depth study of mitochondrial biology with special consideration of the influence of insulin resistance on inter-organ crosstalk will continue to provide a rich substrate for deeper understanding into the common pathobiological mechanisms of chronic diseases and yield new therapeutic opportunities.

## Author Contributions

JS produced the initial draft and figure, MC further edited, provided additional content and references, ET critically reviewed the manuscript. All authors contributed to the article and approved the submitted version.

## Conflict of Interest

The authors declare that the research was conducted in the absence of any commercial or financial relationships that could be construed as a potential conflict of interest.

## Publisher’s Note

All claims expressed in this article are solely those of the authors and do not necessarily represent those of their affiliated organizations, or those of the publisher, the editors and the reviewers. Any product that may be evaluated in this article, or claim that may be made by its manufacturer, is not guaranteed or endorsed by the publisher.
